# Supplementary material for: Deletion of wfs1 Impairs Oligodendrocyte Precursor Cells Dorsal Distribution and Myelination Through the wfs1-hmgcs1 Axis in Zebrafish
Source: Biology (Basel). 2026 Aug 16;15(16):1402. doi: 10.3390/biology15161402 (PMC13510151; doi:10.3390/biology15161402)
Supplement: Supplementary file 1 [file biology-15-01402-s001.zip › File S1-Transcriptome sequencing data and differentially expressed genes (DEGs) in wfs1-- zebrafish at 5 dpf/2.KEGG/wfs1KOvsWT/DOWN/wfs1KOvsWT.down_KEGGdot.pdf]

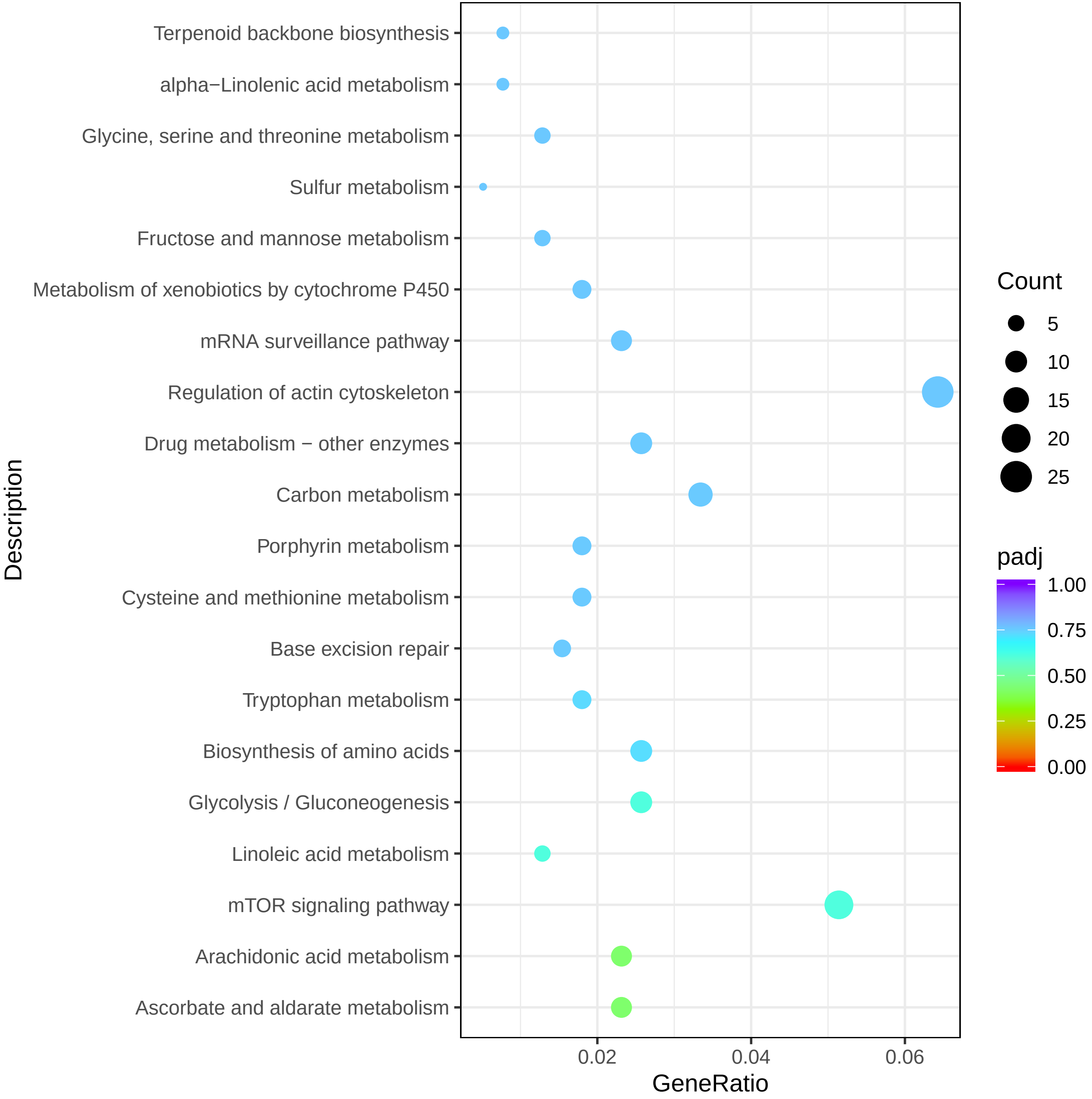

Terpenoid backbone biosynthesis
alpha−Linolenic acid metabolism
Glycine, serine and threonine metabolism
Sulfur metabolism
Fructose and mannose metabolism
Metabolism of xenobiotics by cytochrome P450
mRNA surveillance pathway
Regulation of actin cytoskeleton
Drug metabolism − other enzymes
Carbon metabolism
Porphyrin metabolism
Cysteine and methionine metabolism
Base excision repair
Tryptophan metabolism
Biosynthesis of amino acids
Glycolysis / Gluconeogenesis
Linoleic acid metabolism
mTOR signaling pathway
Arachidonic acid metabolism
Ascorbate and aldarate metabolism
Description
0.02
0.04
0.06
GeneRatio
Count
5
10
15
20
25
padj
1.00
0.75
0.50
0.25
0.00
